# Associations Between Workday/Leisure Day Lifestyle Behavior and Cardiovascular Disease Risk Factors Among Night Shift Workers Using the Isotemporal Substitution Model

**DOI:** 10.3390/healthcare13080908

**Published:** 2025-04-15

**Authors:** Yoko Umeda, Keita Kinoshita, Yoshikuni Sugimura, Yichi Yang, Kyi Mar Wai, Yitao Li, Kazushige Ihara

**Affiliations:** 1 Department of Social Medicine, Hirosaki University Graduate School of Medicine, Hirosaki 036-8562, Japan; umeda@totalfit.co.jp (Y.U.); kinoshita.keita@kao.com (K.K.); y.sugimura@hirosaki-u.ac.jp (Y.S.); yangy@hirosaki-u.ac.jp (Y.Y.); 2 Human Healthcare Research Laboratories, Kao Corporation, Tokyo 131-8501, Japan; 3 Department of Human Ecology, School of International Health, Graduate School of Medicine, The University of Tokyo, Tokyo 113-0033, Japan; kyimar@g.ecc.u-tokyo.ac.jp; 4 Department of Rehabilitation Technology, Sichuan Nursing Vocational College, Chengdu 610100, China; 1004037265yetta@gmail.com

**Keywords:** night shift work, cardiovascular disease, sedentary behavior, physical activity, isotemporal substitution model

## Abstract

**Background/Objectives**: Night shift workers (NSWs) are at a high risk of cardiovascular disease (CVD). However, the association between CVD risk factors and lifestyle behavior (sedentary behavior [SB], physical activity [PA], and sleep) is unclear among NSWs. NSWs lead different lifestyles on workdays and leisure days. This study aimed to investigate the association between lifestyle behavior times and CVD risk factors among NSWs during workdays and leisure days using an isotemporal substitution model. **Methods**: This cross-sectional study included 66 male NSWs. Time spent on lifestyle behaviors was obtained using a tri-axial accelerometer and classified into SB, light-intensity PA, moderate-to-vigorous PA (MVPA), and sleep. Lifestyle behavior times were divided into workdays and leisure days. CVD risk factors were determined based on periodic health checkups. An isotemporal substitution model was used to estimate the effect of replacing one lifestyle behavior with another on CVD risk factors. **Results**: The lifestyle behavior times differed between workdays and leisure days. On workdays, reallocating 30 min of SB to light-intensity PA was significantly associated with a lower waist circumference. In addition, reallocating sleep to SB or MVPA was significantly associated with higher triglyceride levels. On leisure days, reallocating SB or sleep time to MVPA was significantly associated with lower aspartate aminotransferase levels. **Conclusions**: Given the difference in the associations between lifestyle behavior times and CVD risk factors among NSWs between workdays and leisure days, NSWs should be mindful of the time spent on SB, PA, and sleep on workdays and leisure days to achieve healthier outcomes.

## 1. Introduction

A night shift worker (NSW) is defined as an individual who works in shifts for “any work performed for a period of seven or more consecutive hours, including the hours between midnight and 5 a.m.”. Numerous shift systems are in operation [1]. A social economy that operates 24 h a day, 7 days a week (24/7) is supported by shift workers, including those who work at night [2,3]. Therefore, society bears the responsibility to protect the health of NSWs.

NSWs have been reported to be at an increased risk of cardiovascular disease (CVD) and mortality [4,5,6]. While CVD is the leading cause of death worldwide [7,8], there is a lack of focus on CVD prevention for NSWs. Sedentary behavior (SB), reduced physical activity (PA), and shorter sleep durations have been found to be associated with an increased risk of CVD among NSWs [9,10,11]. NSWs are less physically active than day workers [9,12] and often receive inadequate amounts of sleep [13]. The NSW populations are biased toward industries with high levels of physical labor, and PA is diverse and inconsistently reported [9,10,12]. The World Health Organization (WHO) identified physical inactivity as the most important behavioral risk factor for CVD, and SB was recommended to be replaced with light physical activity (LPA) or moderate-to-vigorous PA (MVPA) for all adults [14]. The American Heart Association (AHA) recommends improving short sleeping times. Furthermore, the AHA has added sleep to its checklist for improving cardiovascular health as “Life’s Essential 8 [15]”. The Ministry of Health, Labor, and Welfare’s “Sleep Guide for Ministry of Health, Labour and Welfare (2023)” in Japan added “Employment Status (Shift Work) and Sleep Issues” to its reference information on sleep, as poor sleep among shift workers is associated with CVD risk [16]. Appropriate lifestyles with adequate PA and sleep would reduce the risk of CVD in NSWs.

Recently, an increasing number of epidemiologic studies on PA and exercise have utilized the isotemporal substitution (IS) model [17]. Due to the limit on the amount of time people can spend on daily activities, increasing the time spent on one activity reduces the time available for another. Previous studies that did not employ the IS model overlooked this interdependency when examining the influence of the increase in one activity on health. These studies found that more time spent on a particular activity, e.g., PA, is associated with better health; however, spending more time on that activity does not guarantee better health, as the reduction in time for other activities, e.g., sleep or SB, may have opposite effects on health. In contrast, the IS model can replace time for a lifestyle activity with time for another within a limited time period by capturing the increase or decrease in each lifestyle activity and can show the impact of the replacement on the body.

Previous studies involving NSWs have examined the relationship between health and each lifestyle behavior independently, such as PA being beneficial to cardiovascular health [18], with no reports considering the interdependence of lifestyle behaviors. In addition, the work patterns of NSWs require them to switch between day and night activities on both workdays and leisure days, and their social lives differ from those of typical day workers. Health support is needed to provide specific time-of-day activities according to the working schedule of NSWs. However, to our knowledge, few studies have examined the relationship between different lifestyle behaviors and CVD risk factors using the IS model for NSWs’ workday/leisure-day activities separately.

Therefore, this study aimed to examine the association between SB, PA, sleep duration, and CVD risk factors among NSWs separately for workdays and leisure days, using the IS model.

## 2. Materials and Methods

### 2.1. Study Design and Participants

**Figure 1:**
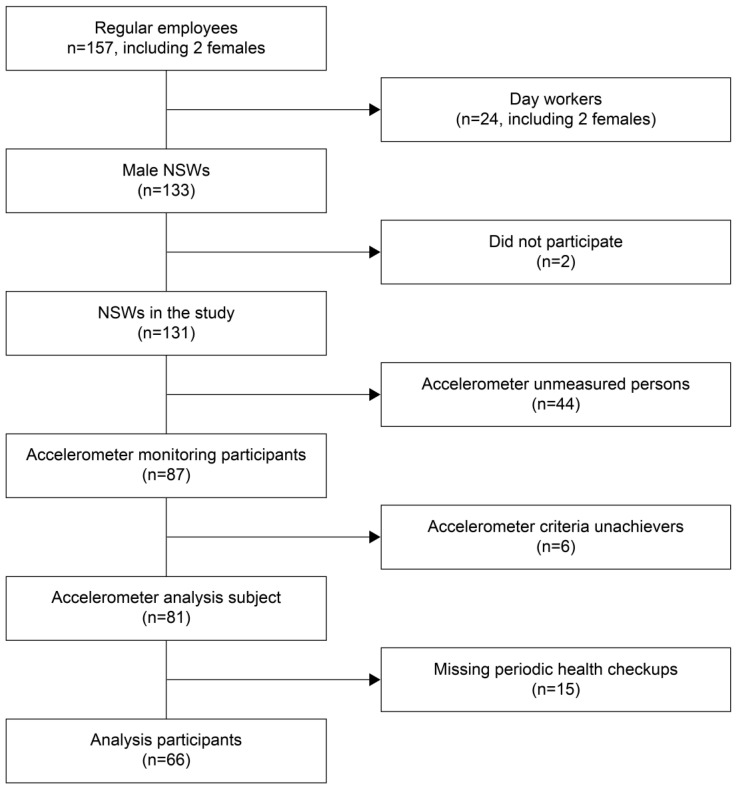
Flowchart of the participant selection process.

This cross-sectional study involved NSWs employed in the Japanese manufacturing industry between September and December 2018. The three manufacturing mills where the participants worked were located in urban areas (business districts separate from residential areas) within the same region (Kansai). The work activities and patterns were uniform across all mills under one company. The potential participants comprised 133 NSWs (all of whom were men), excluding 24 day workers (including two females) from the 157 regular employees of these three manufacturing mills. We excluded two individuals who did not give informed consent, 44 individuals who received an accelerometer but did not wear it, and 15 who did not undergo periodic health examinations at the workplace. Additionally, six workers who wore the accelerometer for less than 3 days were excluded, consistent with previous studies that utilized a similar exclusion criterion [19]. The final analysis included 66 male participants (Figure 1).

This study was approved by the Ethical Review Board of Setsunan University (Osaka, Japan) (approval number: 2018-011) and conducted in accordance with the ethical principles of the Declaration of Helsinki. After receiving an oral explanation of the purpose of the study and the benefits and risks of participation, written informed consent was obtained from the participants. All data were processed anonymously.

### 2.2. Participants’ Work System

**Figure 2:**
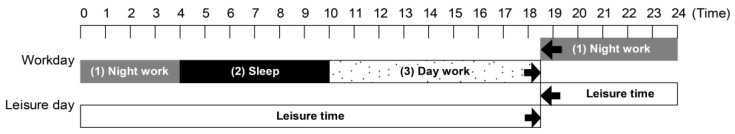
Participant’s work schedule; workday/leisure day times.

The workday was defined as 24 h spent at the company, from arrival to leaving work, consisting of three components: (1) night work, from 18:30 to 04:00 the next day; (2) day work, from 10:00 to 18:30; and (3) free time between (1) and (2), from 04:00 to 10:00 (during which participants could bathe in the company bathroom and sleep in a capsule-type bed). Leisure days were defined as 24 h spent outside the company, from leaving the office until arriving at work the next day (Figure 2).

The basic work pattern involved alternating between work and leisure days, with one consecutive leisure day per week. The company was closed for 1 day per month. The other working days were shift workdays, with the necessary number of employees. The company reported that most employees had used their paid holidays. The number of night shift days (working days) during the implementation period was 10.8 (standard deviation [SD] = 2.0) days/month.

### 2.3. Measurements of SB, PA, and Sleep Duration

SB, PA, and sleep duration were measured using a small triaxial accelerometer (MTN-220, Acos Corporation, Nagano, Japan) weighing 9 g and featuring coin-shaped external dimensions with a diameter of 27 mm and a width of 9.1 mm. The MTN-220 is based on the MTN-210 and FS-750 models, which are known for their reliability and validity in recording daily activities, including sleep [20,21]. These models have the same performance capabilities.

The sampling frequency of the accelerometer was 32 Hz during activity and 8 Hz during sleep, with epoch lengths of 2 s and 2 min, respectively, and the data were recorded continuously for 30 days. The amount of PA discriminated as wakefulness was converted into PA intensities (metabolic equivalents [METs]) and classified as follows: SB was defined as ≤1.5 METs, LPA as 1.6–2.9 METs, and MVPA as ≥3 METs [22,23]. Sleep was cagorized into two types: main sleep, which occurred from bedtime to waking time, and nonmain sleep. In this study, only main sleep was defined as sleep because nonmain sleep, unlike main sleep, may include not only naps or dozing but also a supine stationary posture without actual sleeping.

The participants were instructed to wear the accelerometer on the lower back for 1 week, excluding during bathing. The data collected by the accelerometer were transferred to a personal computer using a reading device, PaSoRi, RC-S300 (SONY Corporation, Tokyo, Japan). The read data were classified into wakefulness and sleep using sleep analysis software (SleepSignAct version 2.0, Kissei Comtec Corporation, Nagano, Japan).

The classification of work and leisure days was confirmed using data obtained from the participants’ work records and accelerometers. The participants wore accelerometers for at least 3 days, including at least 1 workday and 1 leisure day each. The duration of each life activity (SB, LPA, MVPA, and sleep) per workday and leisure day were also calculated. For example, SB time per leisure day was defined as the total SB time for each leisure day divided by the number of leisure days.

### 2.4. CVD Risk Factors

During periodic health examinations at the workplace, blood samples were collected, and serum separation was performed under fasting conditions. This study acquired anthropometric and biochemical measurements data pertaining to the following indices of CVD risk factors: body weight (weight), body mass index (BMI) (weight [kg]/height [m^2^]), waist circumference (WC), blood pressure (systolic blood pressure/diastolic blood pressure), liver function enzyme values (aspartate aminotransferase [AST], alanine aminotransferase [ALT], gamma-glutamyl transpeptidase), cholesterol levels (low-density lipoprotein, high-density lipoprotein [HDL]), and triglycerides (TG) levels. These indices were selected based on items specified in periodic health examinations outlined in the Japanese Occupational Health and Safety Regulations [24]. Alcohol consumption habits were assessed separately on working and leisure days using a self-administered questionnaire. Periodic physical examinations (9 days from 3 September to 17 October 2018) and accelerometry (3 months from 3 September 2018 to 5 December 2018) were initiated on the same day and conducted sequentially according to individual work duties.

### 2.5. Statistical Analysis

The participant characteristics were tested for normality using the Shapiro–Wilk test. Liver function enzyme and triglyceride levels were log-transformed. The means and SDs of measured items were calculated, and the data are shown as means (SDs) unless otherwise noted. Alcohol consumption habits are indicated as numbers and percentages. Comparisons between workdays and leisure days for each lifestyle behavior were performed using the Wilcoxon signed-rank test.

The association between each lifestyle behavior and each CVD risk factor was analyzed using multiple regression analysis with single factors and IS models separately for workdays and leisure days. The dependent variable was one of the 11 CVD risk factors, and the covariates were age, alcohol consumption status, and total lifestyle behavior times.

The IS model was used to quantify the associations of reallocating 30 min of one lifestyle behavior to another with each CVD risk factor (e.g., replacing SB with LPA, MVPA, or sleep by taking SB out of the model). We adopted the 30 min time allocation from the first study that introduced the IS model to medicine [17], as many studies followed the same time unit of allocation [25,26,27], and this ensures comparability of effects among studies. The resulting regression coefficient represented the association between reallocating 30 min of SB to LPA, MVPA, or sleep. For example, the SB can be set as the reference, and the following IS model can be constructed:

CVD risk factor = LPA*β*_1_ + MVPA*β*_2_ + Sleep*β*_3_ + total lifestyle behavior*β*_4_ + Covariates***β***

This equation can also be written as follows:

CVD risk factor = LPA(*β*_1_ +*β*_4_) + MVPA(*β*_2_ +*β*_4_) + Sleep(*β*_3_ +*β*_4_) + SB*β*_4_ + Covariates***β***

The total lifestyle behavior, sleep time, and type of PA, except SB, were set as the independent variables. The regression coefficient *β*_4_ of the total lifestyle behavior demonstrates the numeric association between the reference (SB in the model above) and the corresponding CVD risk factor. The coefficients of LPA, MVPA, and sleep (*β*_1_, *β*_2_, and *β*_3_) show how the value of the CVD risk factor changes when SB is replaced with these lifestyle behaviors. In other words, the model shows how switching from SB to PA or sleep would affect the CVD risk factor.

The single-factor model was used to quantify the associations of 30 min of each lifestyle behavior with each CVD risk factor separately, without considering other behaviors (e.g., SB).

CVD risk factor = SB*β*_1_ + total lifestyle behavior*β*_2_ + Covariates***β***

where *β* denotes the regression coefficients and the total lifestyle behavior is the summation of SB, LPA, MVPA, and sleep time.

Total lifestyle behavior = SB + LPA + MVPA + Sleep

Thus, the structure of the regression equation is equivalent to the following:

CVD risk factor = SB(*β*_1_ +*β*_2_) + LPA*β*_2_ + MVPA*β*_2_ + Sleep*β*_2_ + Covariates***β***

How the SB affects the value of the CVD risk factor can be evaluated based on the summation of regression coefficients of the SB and the total lifestyle behavior (*β*_1_ +*β*_2_).

Multicollinearity among variables in the IS model was evaluated using the variance inflation factor (VIF). A VIF of <5 was considered acceptable. All statistical analyses were conducted using IBM SPSS Statistics for Windows, version 27 (IBM Corp., Armonk, NY, USA). Statistical significance was set at *p* < 0.05.

## 3. Results

Table 1 presents the characteristics of the participants. The mean (SD) age was 40.2 (9.9) years. The accelerometers were worn for an average of 6.2 (1.3) days. Of these, the average number of working and leisure days was 1.9 (0.6) and 4.2 (1.1), respectively.

**Figure 3:**
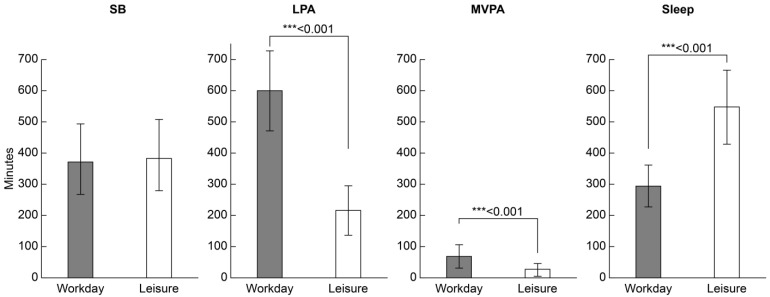
Comparison of daily activity time (minutes/day) for each workday and leisure day. Values are presented as means (standard deviations). Wilcoxon signed-rank test, *** *p* < 0.001. LPA, low-intensity physical activity; MVPA, moderate-to-vigorous physical activity; SB, sedentary behavior.

Figure 3 shows the comparison of the duration of each daily activity on workdays and leisure days. The average LPA on workdays was 599.3 (127.6) min and was significantly longer (*p* < 0.001) than that on leisure days (215.0 [79.8] min). MVPA on workdays (68.9 [37.8] min) was significantly longer than that on leisure days (28.6 [21.6] min) (*p* < 0.001). The sleep duration on workdays (294.7 [68.4] min) was significantly shorter than on leisure days (549.3 [119.3] min) (*p* < 0.001). The SB duration was not significantly different between workdays (372.2 [124.2] min) and leisure days (383.8 [104.2] min).

**Figure 4:**
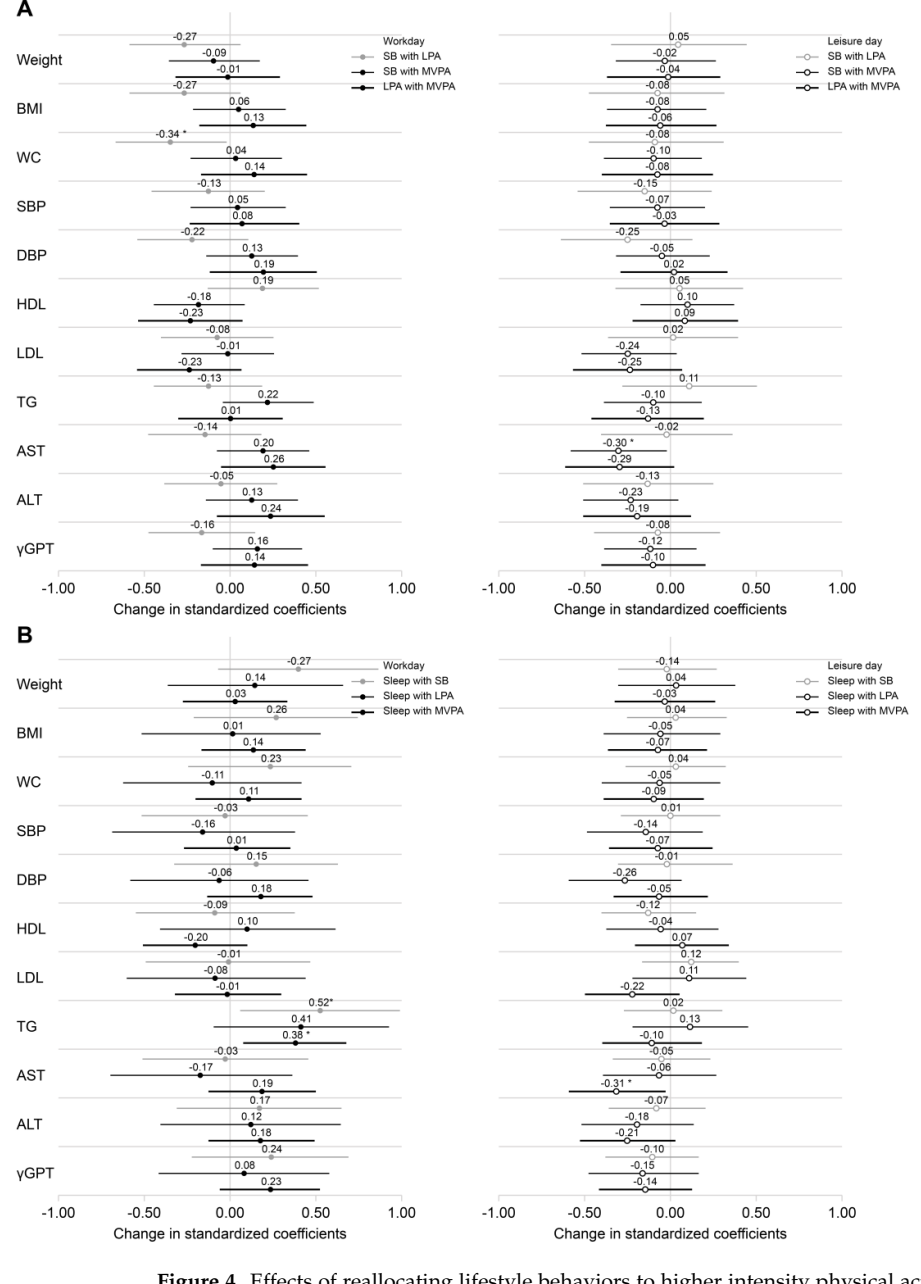
Effects of reallocating lifestyle behaviors to higher intensity physical activity on cardiovascular disease risk factors using the isotemporal substitution model. (**A**) Association of CVD risk factors with SB and LPA replaced by 30 min of higher-intensity PA each. (**B**) Association of CVD risk factors with lifestyle behaviors (SB, LPA, MVPA) replacing sleep for 30 min each. All associations were analyzed separately for workdays and leisure days using the isotemporal substitution model. logALT, logAST, logγ-GPT, and logTG were log-transformed. The covariates included age, alcohol consumption habits, and duration of daily activities. The standardized coefficients for each independent variable represent the change in the outcome variable reassigned from 30 min of lifestyle behaviors to higher-intensity lifestyle behaviors. Values are shown as standardized coefficients (95% confidence intervals). * *p* < 0.05. ALT, alanine aminotransferase; AST, aspartate aminotransferase; CVD, cardiovascular disease; LPA, low-intensity physical activity; MVPA, moderate-to-vigorous physical activity; PA, physical activity; SB, sedentary behavior; TG, triglycerides.

Analysis of the association of each lifestyle behavior of NSWs with CVD risk factors separately for work and leisure days showed different results. Figure 4A and Table S1 show the results of replacing SB and LPA with higher-intensity PA, while Figure 4B and Table S2 show the results of replacing sleep with higher-intensity lifestyle behaviors (SB, LPA, and MVPA), analyzed separately for workdays and leisure days.

On workdays, reallocating 30 min of SB to LPA was significantly associated with a decrease in waist circumference (*β* = −0.344, 95% confidence interval [CI]: −0.668 to −0.019), but was not significantly associated with weight or BMI. In addition, reallocating sleep to SB or MVPA was significantly associated with higher TG levels (sleep to SB, *β* = 0.524, 95% CI: 0.062–0.986; sleep to MVPA, *β* = 0.379, 95% CI: 0.087–0.671). On leisure days, reallocating SB or sleep to MVPA was significantly associated with lower AST levels (SB to MVPA, *β* = −0.298, 95% CI: −0.574 to −0.022; sleep to MVPA, *β* = −0.308, 95% CI: −0.583 to −0.032).

Table S3 presents the association between each lifestyle behavior (30 min) and each CVD risk factor, analyzed using single-factor models separately for workdays and leisure days. Longer SB was significantly associated with higher weight and WC, and longer sleep duration was significantly associated with lower TG levels on workdays. On leisure days, longer MVPA was significantly associated with lower AST and ALT levels.

## 4. Discussion

This study examined the associations of SB, PA, and sleep duration with CVD risk factors among NSWs during workdays and leisure days in the manufacturing industry, utilizing the IS model. On workdays, replacing SB with LPA resulted in a lower WC. Furthermore, replacing sleep with SB or MVPA resulted in higher TG levels. Leisure days were associated with lower AST levels when SB and sleep were replaced with MVPA (Table 2).

To our knowledge, this is the first study to apply the IS model to the analysis of PA among NSWs. Furthermore, it is the first to analyze the relationship between PA and CVD risk factors among NSWs separately for workdays and leisure days. A previous study examined the association between PA and CVD risk factors among blue-collar workers (15% NSWs and 79% fixed-day shift workers). The study reported that reallocating sedentary time to higher-intensity PA was associated with lower obesity indicators in both work and leisure time on workdays [28]. In contrast, the present study focused exclusively on NSWs, splitting their days into workdays and leisure days. The study showed that the association between lifestyle behaviors, including PA, and CVD risk factors varied between workdays and leisure days. This suggests that adequate PA and/or sleep is recommended on both workdays and leisure days.

Workday PA was associated with a lower WC when SB was replaced with LPA with the support of the positive association of SB with WC and weight in the single-factor models. Previous studies have found that replacing SB with LPA or MVPA was associated with a reduced risk of obesity in the general adult population [29,30]. Replacing SB with LPA also has the potential to reduce the risk of obesity in NSWs. Sleep on workdays was associated with higher TG levels due to the substitution of SB and MVPA in the present study. Additionally, sleep was negatively associated with TG levels in the single-factor model. Elevated TG levels due to short sleep durations have been reported in several studies involving the general adult population (non-IS models) [29,31,32,33]. Shorter sleep may disrupt lipid metabolism through hormonal changes such as increased cortisol and insulin resistance. Short sleep duration has also been reported to be associated with low serum leptin levels [34]. A meta-analysis has shown that shorter sleep has increased energy intake, accompanied by significantly higher fat intake [35]. Behavioral mechanisms may also be involved while triglyceride has been reported to induce central leptin and insulin resistance [36]. The participants who had shorter sleep on workday in our study may eat store-bought snacks and drink beverages during sleep time allocated in the company. In the general adult population, 7 h of sleep (not short or long) and MVPA have been shown to reduce the risk of CVD [29]. Blodgett et al. [31] found a positive effect of increased MVPA on cardiovascular and metabolic health and higher TG levels with the replacement of MVPA with 7.7 h of sleep. Although this report contradicts our study, Blodgett et al. [31] hypothesized that those who sleep poorly (<6 h) may benefit more from prioritizing sleep over PA. As the participants in the present study also slept poorly on workdays (<5 h), we believe that replacing MVPA with sleep would have a positive impact on TG levels. On workdays, NSWs should limit the amount of time spent being sedentary and incorporate intervals of standing and movement. Additionally, prioritizing adequate sleep following night shifts on workdays is recommended.

On leisure days, reallocating SB or sleep to MVPA was significantly associated with lower AST levels. In the single-factor model, MVPA was negatively associated with AST and ALT levels. AST has been reported to be associated with CVD mortality [37] while AST is not commonly regarded as CVD risk factor. AST and ALT levels are among the diagnostic criteria for non-alcoholic fatty liver disease (NAFLD) [38]. CVD risk factors have reportedly been increasing among patients with NAFLD [39]. A higher AST in the present study may reflect liver dysfunction or fibrosis potentially related to NAFLD. In 2023, NAFLD was renamed to metabolic dysfunction-associated steatotic liver disease (MASLD) [40]. Therefore, liver disease reports are mixed in terms of the utilization of the terms NAFLD and MASLD. NAFLD treatment guidelines [41] acknowledge that PA can lead to a reduction in ALT and AST levels, thereby improving NAFLD. These guidelines advocate for a direct PA regimen consisting of 30–60 min of exercise 3–5 days per week [42]. Increased PA was reportedly associated with a lower CVD risk among patients with NAFLD [43]. Targher et al. [44] suggested moderate exercise for ≥150 min/week or intense exercise for ≥75 min/week for CVD risk reduction in patients with MASLD, as MASLD is an independent risk factor for CVD morbidity and mortality. MVPA on leisure days may lower AST and ALT levels and reduce CVD risk due to hepatic adipose tissue. Previous cross-sectional and longitudinal studies have reported an association between longer sleep durations and NAFLD. These studies suggest that a longer sleep duration decreases energy expenditure as a consequence of the decreased amount of time spent awake, metabolic disturbances, and/or as a consequence of behavioral changes [45,46]. Substituting excessive sleep and SB with MVPA, such as walking, hiking, climbing stairs, and running, on leisure days may mitigate CVD risk factors.

Taking the results and discussion mentioned above into account, we suggest practical recommendations to decrease CVD risk for NSWs. On workdays, NSWs should increase their sleep time to more than 6 h, which corresponds to the recommended sleep time (at least 6 h) for Japanese general population [16], reducing SB or MVPA. For those who have secured enough sleep time, we recommend replacing SB with LPA at least 10 min, which is in accordance with a physical activity guideline for the Japanese general population [47], e.g., exercising or walking for 10 min during lunch time or breaks. Behavioral change cannot be achieved through individual efforts of NSWs alone and needs multi-level supports. Especially, reducing SB or MVPA on workdays needs negotiation with employers. We should accumulate pieces of evidence on lifestyle activities so that the evidence would be reflected to upgrading the guidelines to secure reducing SB and increasing sleep time for NSWs. On leisure days, we recommend that NSWs increase MVPA to more than 60 min per day by substituting SB or sleep time with more engaging activities like walking and cycling. This recommendation may be achieved by motivational intervention provided by health professionals, with adjustment to meet country-specific guidelines. Motivational intervention would also be useful for lifestyle changes in workdays. NSWs can set a goal to go to sleep soon after entering the bedroom without eating or chatting, which may help reduce SB, even on days when they are at their workplace. We should note that the recommendations are based on the findings of the present study. We need further studies with a large sample including women and workers form different industries.

In terms of the study’s strengths, the NSWs had consistent 24 h behavior during workdays owing to uniform manufacturing line operations. This inherent consistency enhanced the reliability of the study, obviating the need to adjust for the timing of lifestyle behaviors. Furthermore, the use of accelerometers attached to the belt facilitated continuous wearing and objective data acquisition for SB, PA, and sleep. This method ensured safety by preventing the risk of the accelerometer falling into the manufacturing machine. Additionally, it facilitated the process of changing work attire. While the accelerometer can capture both main sleep and nonmain sleep, sleep time in this has not included nonmain sleep to avoid overestimation of sleep time, as mentioned in the “Methods.” This procedure may lead to an underestimation of sleep time. This might be a limitation of this study. However, we do not know whether this underestimation brought biased results in the association between sleep and CVD risk factors.

This study had several other limitations. First, we did not consider sleep other than primary sleep or sleep quality. Second, the effects of possible confounders including diet or eating habits, medication use for cardiometabolic diseases, smoking, education, income, blood sugar levels, diabetes, chronic inflammation markers, the degree of social opportunities, years of employment, and consecutive night shifts could not be controlled. We did not evaluate genetic factors either, such as apolipoprotein gene and proinflammatory cytokine gene, which are related to CVD. Third, this study only included men employed by a specific company. Gender bias limits generalizability. As most participants were performing the same job in the mills, we could not consider the effects of different workloads and intensities. These mean that we could not consider possible differences in CVD risk factors among different workplaces. Fourth, lower participation rate in the present study prevents us to eliminate the likely healthy worker’s bias; the absolute value of each *β* coefficient might be greater than the obtained one, providing a greater reallocating effect of lifestyle behavior. Fifth, the small sample size might have resulted in the study being underpowered or led to the discovery of significant associations by chance. Future studies with larger and more diverse samples are needed to validate the findings and generalize the results. These studies should include female workers and those with varying workloads, intensities, and shift patterns from different occupations, while controlling for various potential confounders. However, previous studies with large sample sizes often included shift workers with diverse schedules from various companies without specific criteria for shift patterns [12,28]. The diverse shift patterns likely led to variability in the lifestyle behaviors of participants [12]. Different shift patterns likely influenced the association between lifestyles and CVD risk factors. Therefore, different shift patterns make it difficult to decipher how night shift work influences CVD risk factors. A strength of the present study was its use of a sample with homogeneous shift patterns despite its small size. An additional limitation was the cross-sectional design of the present study. A longitudinal investigation is needed. However, this study that adopted the isotemporal substitution model contributes to a better understanding of the associations between workday/leisure day lifestyle behavior and CVD risk factors among NSWs.

## 5. Conclusions

This study, which adopted the isotemporal substitution model and used a triaxial accelerometer, found that lifestyle behaviors and CVD risk factors were differentially related to workdays and leisure days among NSWs. We suggest that NSWs can reduce the risk of CVD by adopting distinct lifestyle behaviors on workdays and leisure days, substituting SB with LPA, prioritizing adequate sleep following night shifts on workdays, and engaging in more activity pursuits by actively incorporating MVPA into their lifestyles on leisure days. Recommendations for NSWs on workdays include exercising for 10 min during breaks and going to bed earlier without distractions like eating or chatting. On leisure days, recommendations include encouraging people to get up early and go hiking. Further intervention studies are required to confirm the effectiveness of the suggestions. Our method of dividing workdays and leisure days to study the impact of night shift work on health should be extended to other studies.

## Figures and Tables

**Table 1:** Participant characteristics.

		Mean	SD
Age (years)		40.2	9.9
Weight (kg)		69.5	9.7
BMI (kg/m^2^)		23.7	3.5
WC (cm)		84.8	9.3
SBP (mmHg)		121.9	13.0
DBP (mmHg)		75.2	9.6
HDL (mg/dL)		55.7	14.2
LDL (mg/dL)		115.4	27.4
logTG		2.0	0.3
logAST		1.4	0.1
logALT		1.5	0.2
logγGPT		1.7	0.3
Number of accelerometer-wearing days		6.2	1.3
Number of workdays		1.9	0.6
Number of leisure days		4.2	1.1
Alcohol consumption status		n	%
	Drinker	39	59
	Non-drinker	27	41

ALT, alanine aminotransferase; AST, aspartate aminotransferase; BMI, body mass index; γGPT, γ-glutamyltransferase; DBP, diastolic blood pressure; HDL, high-density lipoprotein; LDL, low-density lipoprotein; SBP, systolic blood pressure; TG, triglycerides; WC, waist circumference. Log transformation was performed: logTG, logAST, logALT, logγ-GPT. Drinking data are presented as the number of individuals (n) and as a percentage (rate).

**Table 2:** A summarized table for the effects of reallocating lifestyle behaviors on cardiovascular disease risk factors for workdays and leisure days, as identified by the isotemporal substitution model.

Workdays				
Lifestyle Behaviors	Reallocation	WC	AST	TG
SB	LPA	**↓**	ns	ns
Sleep	SB	ns	ns	**↑**
Sleep	MVPA	ns	ns	**↑**
**Leisure days**				
SB	MVPA	ns	**↓**	ns
Sleep	MVPA	ns	**↓**	ns

The up and down arrows represent the changes in outcome variables resulting from reallocating 30 min of various lifestyle behaviors to high- or low-intensity physical activities. An upward-facing arrow (↑) indicates an increase, while a downward-facing arrow (↓) indicates a decrease in the value of the outcome after reallocation. An “ns” indicates that no significant reallocating effect was found. WC, waist circumference; AST, aspartate aminotransferase; TG, triglycerides; SB, sedentary behavior; LPA, low-intensity physical activity; MVPA, moderate-to-vigorous physical activity; AST and TG were analyzed after log transformation. The models were adjusted for covariates of age, alcohol consumption habits, and duration of daily activities.

## Data Availability

The original contributions presented in this study are included in the article/Supplementary Material. Further inquiries can be directed to the corresponding author.

## Supplementary Materials

The following supporting information can be downloaded at https://www.mdpi.com/article/10.3390/healthcare13080908/s1. Table S1: Association of CVD risk factors with SB and LPA replaced with high-intensity PA for 30 min each during workdays and leisure days (IS model); Table S2: Association with CVD risk factors when sleep was replaced by higher-intensity lifestyle behaviors (SB, LPA, MVPA) for 30 min each during workdays and leisure days (IS model); Table S3: Association between each lifestyle behavior (30 min) and each CVD risk factor on workdays and leisure days (single-factor model).

## Abbreviations

The following abbreviations are used in this manuscript:

NSW	Night shift worker
CVD	Cardiovascular disease
SB	Sedentary behavior
PA	Physical activity
MVPA	Moderate-to-vigorous PA
