# Supplementary material for: Associations Between Workday/Leisure Day Lifestyle Behavior and Cardiovascular Disease Risk Factors Among Night Shift Workers Using the Isotemporal Substitution Model
Source: Healthcare (Basel). 2025 Apr 15;13(8):908. doi: 10.3390/healthcare13080908 (PMC12026880; doi:10.3390/healthcare13080908)
Supplement: Supplementary file 1 [file healthcare-13-00908-s001.zip › healthcare-3542630-supplementary.pdf]

Supplementary Materials

Table S1. Association of CVD risk factors with SB and LPA replaced with high-intensity PA for 30 minutes each during workdays and leisure days (IS model).

	SB with LPA				SB with MVPA				LPA with MVPA			
	95%CI				95%CI				95%CI			
**Workdays**	β	Lower	Upper	p-value	β	Lower	Upper	p-value	β	Lower	Upper	p-value
Weight (kg)	-0.266	-0.585	0.054	0.101	-0.093	-0.357	0.172	0.486	-0.014	-0.315	0.288	0.927
BMI (kg/m²)	-0.265	-0.589	0.059	0.108	0.055	-0.213	0.324	0.682	0.134	-0.172	0.440	0.385
WC (cm)	-0.344	-0.668	-0.019	**0.038**	0.038	-0.231	0.306	0.780	0.140	-0.167	0.446	0.365
SBP (mmHg)	-0.126	-0.458	0.206	0.450	0.047	-0.228	0.322	0.735	0.084	-0.229	0.398	0.592
DBP (mmHg)	-0.218	-0.542	0.107	0.185	0.130	-0.139	0.398	0.338	0.194	-0.112	0.501	0.209
HDL (mg/dL)	0.193	-0.125	0.510	0.229	-0.175	-0.438	0.087	0.187	-0.233	-0.532	0.067	0.126
LDL (mg/dL)	-0.075	-0.400	0.250	0.646	-0.011	-0.280	0.258	0.937	-0.233	-0.532	0.067	0.126
logTG	-0.127	-0.442	0.189	0.425	0.220	-0.042	0.481	0.098	0.012	-0.295	0.318	0.940
logAST	-0.144	-0.474	0.187	0.388	0.195	-0.079	0.469	0.159	0.258	-0.040	0.555	0.089
logALT	-0.054	-0.380	0.272	0.742	0.127	-0.142	0.397	0.349	0.238	-0.074	0.550	0.133
logγGPT	-0.162	-0.472	0.147	0.298	0.160	-0.097	0.416	0.219	0.143	-0.164	0.451	0.354
**Leisure Days**												
Weight (kg)	0.052	-0.343	0.447	0.793	-0.024	-0.311	0.263	0.867	-0.038	-0.360	0.284	0.813
BMI (kg/m²)	-0.077	-0.470	0.315	0.695	-0.078	-0.363	0.207	0.587	-0.057	-0.376	0.263	0.723
WC (cm)	-0.081	-0.473	0.310	0.679	-0.098	-0.383	0.186	0.492	-0.076	-0.395	0.242	0.634
SBP (mmHg)	-0.147	-0.531	0.238	0.449	-0.071	-0.351	0.208	0.612	-0.031	-0.345	0.282	0.842

DBP (mmHg)	-0.251	-0.63	0.129	0.192	-0.045	-0.321	0.231	0.744	0.023	-0.286	0.332	0.883
HDL (mg/dL)	0.051	-0.321	0.423	0.786	0.099	-0.171	0.369	0.465	0.085	-0.217	0.388	0.574
LDL (mg/dL)	0.018	-0.361	0.397	0.924	-0.241	-0.517	0.034	0.085	-0.246	-0.555	0.062	0.116
logTG	0.111	-0.282	0.504	0.574	-0.100	-0.386	0.185	0.484	-0.131	-0.451	0.189	0.416
logAST	-0.021	-0.401	0.359	0.912	-0.298	-0.574	-0.022	**0.035**	-0.292	-0.602	0.017	0.064
logALT	-0.129	-0.507	0.249	0.497	-0.229	-0.504	0.046	0.101	-0.194	-0.501	0.114	0.213
logγGPT	-0.075	-0.440	0.289	0.681	-0.117	-0.382	0.148	0.379	-0.097	-0.393	0.200	0.518

ALT, alanine aminotransferase; AST, aspartate aminotransferase; BMI, body mass index; γGPT, γ-glutamyltransferase; CI, confidence interval; CVD, cardiovascular disease; DBP, diastolic blood pressure; HDL, high-density lipoprotein; IS, isotemporal substitution; LDL, low-density lipoprotein; LPA, low-intensity physical activity; MVPA, moderate-to-vigorous physical activity; PA, physical activity; SB, sedentary behavior; SBP, systolic blood pressure; TG, triglycerides; WC, waist circumference. Boldface indicates $p<0.05$.

Table S2. Association with CVD risk factors when sleep was replaced by higher-intensity lifestyle behaviors (SB, LPA, MVPA) for 30 minutes each during workdays and leisure day (IS model).

**Workdays**	Sleep with SB				Sleep with LPA				Sleep with MVPA			
		95%CI				95%CI				95%CI		
	β	Lower	Upper	p-value	β	Lower	Upper	p-value	β	Lower	Upper	p-value
Weight (kg)	0.397	-0.070	0.864	0.094	0.142	-0.369	0.653	0.580	0.028	-0.267	0.324	0.850
BMI (kg/m²)	0.264	-0.211	0.738	0.271	0.006	-0.512	0.524	0.982	0.135	-0.164	0.435	0.370
WC (cm)	0.232	-0.243	0.707	0.331	-0.105	-0.624	0.414	0.686	0.108	-0.192	0.409	0.473
SBP (mmHg)	-0.032	-0.518	0.454	0.896	-0.159	-0.690	0.372	0.551	0.037	-0.270	0.344	0.811
DBP (mmHg)	0.150	-0.325	0.625	0.530	-0.064	-0.583	0.455	0.806	0.175	-0.125	0.475	0.248
HDL (mg/dL)	-0.090	-0.554	0.375	0.701	0.101	-0.407	0.608	0.693	-0.203	-0.496	0.091	0.172
LDL (mg/dL)	-0.009	-0.484	0.466	0.970	-0.084	-0.604	0.435	0.747	-0.013	-0.314	0.287	0.929
logTG	0.524	0.062	0.986	**0.027**	0.412	-0.093	0.916	0.108	0.379	0.087	0.671	**0.012**
logAST	-0.029	-0.513	0.455	0.904	-0.174	-0.702	0.355	0.513	0.186	-0.120	0.492	0.229
logALT	0.169	-0.308	0.646	0.481	0.120	-0.401	0.641	0.647	0.179	-0.123	0.480	0.240
logγGPT	0.237	-0.217	0.690	0.300	0.081	-0.415	0.576	0.745	0.232	-0.055	0.518	0.111
**Leisure Days**												
Weight (kg)	-0.011	-0.305	0.283	0.941	0.044	-0.298	0.385	0.799	-0.026	-0.313	0.260	0.855
BMI (kg/m²)	0.040	-0.251	0.332	0.782	-0.046	-0.386	0.293	0.785	-0.069	-0.354	0.215	0.627
WC (cm)	0.039	-0.252	0.330	0.791	-0.052	-0.390	0.287	0.761	-0.090	-0.375	0.194	0.527
SBP (mmHg)	0.008	-0.277	0.294	0.953	-0.140	-0.473	0.192	0.402	-0.069	-0.349	0.21	0.620
DBP (mmHg)	-0.011	-0.293	0.271	0.940	-0.259	-0.587	0.069	0.120	-0.048	-0.323	0.228	0.731

HDL (mg/dL)	-0.121	-0.398	0.155	0.384	-0.042	-0.364	0.280	0.795	0.074	-0.196	0.344	0.585
LDL (mg/dL)	0.124	-0.157	0.406	0.381	0.113	-0.214	0.441	0.491	-0.215	-0.491	0.060	0.122
logTG	0.021	-0.271	0.313	0.887	0.127	-0.213	0.467	0.457	-0.096	-0.381	0.189	0.503
logAST	-0.045	-0.327	0.238	0.752	-0.056	-0.384	0.273	0.736	-0.308	-0.583	-0.032	**0.029**
logALT	-0.072	-0.352	0.209	0.612	-0.184	-0.511	0.143	0.264	-0.244	-0.518	0.031	0.081
logγGPT	-0.098	-0.369	0.173	0.470	-0.151	-0.466	0.164	0.342	-0.138	-0.402	0.127	0.302

ALT, alanine aminotransferase; AST, aspartate aminotransferase; BMI, body mass index; γGPT, γ-glutamyltransferase; CI, confidence interval; CVD, cardiovascular disease; DBP, diastolic blood pressure; HDL, high-density lipoprotein; IS, isotemporal substitution; LDL, low-density lipoprotein; LPA, low-intensity physical activity; MVPA, moderate-to-vigorous physical activity; PA, physical activity; SB, sedentary behavior; SBP, systolic blood pressure; TG, triglycerides; WC, waist circumference. logTG, logAST, logALT, and logγ-GPT were log-transformed. Covariates: age, alcohol consumption, and duration of daily activities. The standardized coefficients for each independent variable represent the change in the outcome variable resulting from reallocating 30 minutes of lifestyle behavior time to higher-intensity lifestyle behaviors. Values are presented as β (unstandardized coefficient) (95% confidence interval). Boldface indicates $p<0.05$

Table S3. Association between each lifestyle behavior (30 minutes) and each CVD risk factor on workdays and leisure days (single-factor model).

	SB				LPA				MVPA				Sleep			
		95%CI				95%CI				95%CI				95%CI		
**Workdays**	β	Lower	Upper	p-value	β	Lower	Upper	p-value	β	Lower	Upper	p-value	β	Lower	Upper	p-value
Weight (kg)	0.675	0.096	1.254	**0.023**	-0.518	-1.207	0.171	0.138	-0.986	-2.985	1.014	0.328	-0.653	-1.725	0.420	0.228
BMI (kg/m²)	0.166	-0.046	0.379	0.123	-0.168	-0.416	0.081	0.182	0.034	-0.690	0.758	0.925	-0.145	-0.533	0.243	0.457
WC (cm)	0.569	0.001	1.138	**0.0495**	-0.644	-1.306	0.017	0.056	-0.179	-2.138	1.780	0.855	-0.244	-1.297	0.809	0.645
SBP (mmHg)	0.181	-0.628	0.990	0.657	-0.359	-1.296	0.577	0.446	0.177	-2.526	2.881	0.896	0.226	-1.229	1.681	0.757
DBP (mmHg)	0.260	-0.329	0.848	0.381	-0.347	-1.029	0.335	0.313	0.687	-1.281	2.655	0.488	-0.208	-1.271	0.855	0.697
HDL (mg/dL)	-0.220	-1.081	0.641	0.612	0.418	-0.578	1.414	0.405	-1.571	-4.422	1.280	0.275	0.164	-1.386	1.714	0.833
LDL (mg/dL)	0.356	-1.312	2.023	0.671	-0.515	-2.450	1.419	0.596	-0.593	-6.162	4.976	0.832	0.258	-2.742	3.257	0.864
logTG	0.006	-0.014	0.026	0.564	0.002	-0.021	0.025	0.871	0.056	-0.009	0.121	0.090	-0.039	-0.074	-0.005	**0.026**
logAST	0.000	-0.009	0.009	0.975	-0.003	-0.013	0.007	0.565	0.017	-0.012	0.046	0.234	0.002	-0.014	0.017	0.845
logALT	0.000	-0.014	0.015	0.953	0.001	-0.016	0.017	0.927	0.023	-0.025	0.07	0.341	-0.010	-0.035	0.016	0.447
logγGPT	0.006	-0.013	0.026	0.532	-0.006	-0.029	0.017	0.601	0.036	-0.029	0.101	0.272	-0.016	-0.051	0.019	0.366
**Leisure Days**																
Weight (kg)	-0.032	-0.828	0.764	0.936	0.135	-1.045	1.314	0.820	-0.221	-3.875	3.434	0.904	-0.014	-0.689	0.662	0.968
BMI (kg/m²)	0.054	-0.232	0.339	0.709	-0.094	-0.517	0.329	0.659	-0.408	-1.717	0.901	0.535	0.006	-0.237	0.249	0.960
WC (cm)	0.147	-0.616	0.910	0.702	-0.290	-1.419	0.840	0.610	-1.375	-4.864	2.115	0.434	0.036	-0.612	0.684	0.911
SBP (mmHg)	0.110	-0.941	1.160	0.835	-0.789	-2.333	0.755	0.311	-1.804	-6.607	2.998	0.455	0.241	-0.648	1.130	0.590
DBP (mmHg)	0.049	-0.726	0.825	0.899	-0.978	-2.10	0.144	0.086	-1.343	-4.888	2.201	0.451	0.331	-0.322	0.984	0.315

HDL (mg/dL)	-0.519	-1.624	0.585	0.351	-0.033	-1.681	1.615	0.968	1.562	-3.529	6.653	0.542	0.331	-0.609	1.271	0.484
LDL (mg/dL)	1.123	-1.088	3.334	0.314	0.362	-2.941	3.665	0.827	-7.860	-17.897	2.176	0.123	-0.659	-2.543	1.226	0.487
logTG	0.002	-0.024	0.028	0.880	0.012	-0.027	0.050	0.551	-0.031	-0.150	0.088	0.603	-0.004	-0.026	0.018	0.709
logAST	0.000	-0.011	0.012	0.994	-0.008	-0.024	0.009	0.379	-0.061	-0.112	-0.010	**0.019**	0.005	-0.005	0.014	0.355
logALT	-0.002	-0.021	0.018	0.874	-0.022	-0.050	0.006	0.126	-0.088	-0.173	-0.003	**0.042**	0.011	-0.005	0.027	0.167
logγGPT	-0.006	-0.032	0.019	0.624	-0.023	-0.060	0.015	0.237	-0.073	-0.190	0.044	0.215	0.014	-0.007	0.036	0.185

ALT, alanine aminotransferase; AST, aspartate aminotransferase; BMI, body mass index; γGPT, γ-glutamyltransferase; CI, confidence interval; CVD, cardiovascular disease; DBP, diastolic blood pressure; HDL, high-density lipoprotein; IS, isotemporal substitution; LDL, low-density lipoprotein; LPA, low-intensity physical activity; MVPA, moderate-to-vigorous physical activity; PA, physical activity; SB, sedentary behavior; SBP, systolic blood pressure; TG, triglycerides; WC, waist circumference. logTG, logAST, logALT, and logγ-GPT were log-transformed. Covariates: Age, alcohol consumption, and duration of daily activities.

Values are shown as β (unstandardized coefficient) (95% confidence interval). Boldface indicates $p<0.05$.
